# Intraspecific diversification of the crop wild relative *Brassica cretica* Lam*.* using demographic model selection

**DOI:** 10.1186/s12864-019-6439-x

**Published:** 2020-01-14

**Authors:** Antonios Kioukis, Vassiliki A. Michalopoulou, Laura Briers, Stergios Pirintsos, David J. Studholme, Pavlos Pavlidis, Panagiotis F. Sarris

**Affiliations:** 1 Institute of Molecular Biology and Biotechnology, Foundation for Research and Technology-Hellas, Heraklion, 70013 Crete, Greece; 2 Biosciences, College of Life and Environmental Sciences, University of Exeter, Exeter, UK; 3 Institute of Computer Science, Foundation for Research and Technology-Hellas, Heraklion, 70013 Crete, Greece; 4 Department of Biology, University of Crete, 714 09 Heraklion, Greece; 5 Botanical Garden, University of Crete, Gallos Campus, 741 00 Rethymnon, Greece

**Keywords:** *Brassica cretica* Lam., *Brassica oleracea*, Crop wild relatives, Draft genome, de novo sequencing

## Abstract

**Background:**

Crop wild relatives (CWRs) contain genetic diversity, representing an invaluable resource for crop improvement. Many of their traits have the potential to help crops to adapt to changing conditions that they experience due to climate change. An impressive global effort for the conservation of various CWR will facilitate their use in crop breeding for food security.

The genus *Brassica* is listed in Annex I of the International Treaty on Plant Genetic Resources for Food and Agriculture. *Brassica oleracea* (or wild cabbage), a species native to southern and western Europe, has become established as an important human food crop plant because of its large reserves stored over the winter in its leaves.

*Brassica cretica* Lam. (*Bc*) is a CWR in the brassica group and *B. cretica* subsp. *nivea* (*Bcn*) has been suggested as a separate subspecies. The species *Bc* has been proposed as a potential gene donor to brassica crops, including broccoli, cabbage, cauliflower, oilseed rape, etc.

**Results:**

We sequenced genomes of four *Bc* individuals, including two *Bcn* and two *Bc*. Demographic analysis based on our whole-genome sequence data suggests that populations of *Bc* are not isolated. Classification of the *Bc* into distinct subspecies is not supported by the data. Using only the non-coding part of the data (thus, the parts of the genome that has evolved nearly neutrally), we find the gene flow between different *Bc* population is recent and its genomic diversity is high.

**Conclusions:**

Despite predictions on the disruptive effect of gene flow in adaptation, when selection is not strong enough to prevent the loss of locally adapted alleles, studies show that gene flow can promote adaptation, that local adaptations can be maintained despite high gene flow, and that genetic architecture plays a fundamental role in the origin and maintenance of local adaptation with gene flow. Thus, in the genomic era it is important to link the selected demographic models with the underlying processes of genomic variation because, if this variation is largely selectively neutral, we cannot assume that a diverse population of crop wild relatives will necessarily exhibit the wide-ranging adaptive diversity required for further crop improvement.

## Background

### Crop wild relatives

Although many plant species are used in food and agriculture, only 30 crops account for the 95% of food production worldwide [1]. Domesticated crops, used for food production, show reduced genetic diversity compared to their respective crop wild relatives (CWRs). This genetic “bottleneck” of domestication [2] resulted in loss of valuable alleles. On the other hand, during the domestication process, introgression from wild species may generate additional genetic diversity [3, 4].

As wild ‘progenitors’ of crops continue to evolve under abiotic and biotic stresses, it is very important to conserve this resulting genetic biodiversity, which can be useful for agriculture (in situ conservation). Seed banks or germplasm collections are also important to preserve as another resource for agriculture (ex situ conservation). The total genome sequencing of CWRs may be used first to characterize wild populations and inform strategy for their conservation. On the other hand, analysis of the sequence can reveal genetic variation and important genetic characters that have been lost during domestication, and that could be transferred into crop species to support food security, climate adaptation and nutritional improvement [1]. The ready availability of low-cost and high-throughput re-sequencing technologies enables the survey of CWR genomes for genetic variation and novel genes and alleles.

Recent decades have seen some remarkable examples of introducing favored traits from CWRs into their respective domesticated crop plants. In most cases, these traits concern resistance to biotic stresses, such as resistance to late blight (*Phytophthora infestans)* from the wild potato *Solanum demissum* Lindl [5, 6].. Besides biotic tolerance, many quantitative trait loci have been identified and/or introduced, affecting the grain quality for increased yield, such as from *Oryza rufipogon*, a wild species of rice, to *Oryza sativa* [7] and grain hardness from *Hordeum spontaneum* (wild barley) [8].

### *Brassica oleracea*: crops and genomic features

*Brassica oleracea* L. belongs to the family *Brassicacea* and is a very important domesticated plant species, comprised of many vegetable crops as different cultivars, such as cauliflower, broccoli, cabbages, kale, Brussels sprouts, savoy, kohlrabi and gai lan. *Brassica oleracea* includes wild cabbage, which is found in coastal southern and western Europe. The species has become very popular because of its high content of nutrients, such as vitamin C, its anticancer properties [9] and the high food reserves in its leaves.

*Brassica oleracea* constitutes one of the three diploid *Brassica* species in the classical triangle of U (Nagaharu U. 1935) [10] (genome: CC), that contains nine chromosomes. The other two species in this group are *B. rapa* (L.) (genome: AA) with 10 chromosomes and *B. nigra* (L.) W. D. J. Koch (the black mustard) (genome: BB) with 8 chromosomes.

These three closely related species gave rise to new allotetraploid species that are very important oilseed crops: *B. juncea* (genome: AABB), *B. napus* L. (genome: AACC) and *B. carinata* (genome: BBCC). There is evidence for each of the *Brassica* genomes having undergone a whole-genome duplication [11, 12] and a *Brassicaceae*-lineage-specific whole-genome triplication, which followed the divergence from the *Arabidopsis* lineage [13, 14].

In 2014, Liu et al. [15] reported a draft genome of *B. oleracea* var. *capitata* and a genomic comparison with its very close sister species *B. rapa*. A total of 45,758 protein-coding genes were predicted, with mean transcript length of 1761 bp and 3756 non-coding RNAs (miRNA, tRNA, rRNA and snRNA). It is observed that there is a greater number of transposable elements (TEs) in *B. oleracea* than in *B. rapa* as a consequence of continuous amplification over the last 4 million years (MY), the time that the two species diverged from a common ancestor, whereas in *B. rapa* the amplification occurred mostly in the last 0.2 MY [15]. Moreover, there has been massive gene loss and frequent reshuffling of triplicated genomic blocks, which favored over-retention of genes for metabolic pathways.

### *Brassica cretica*

**Fig. 1:**
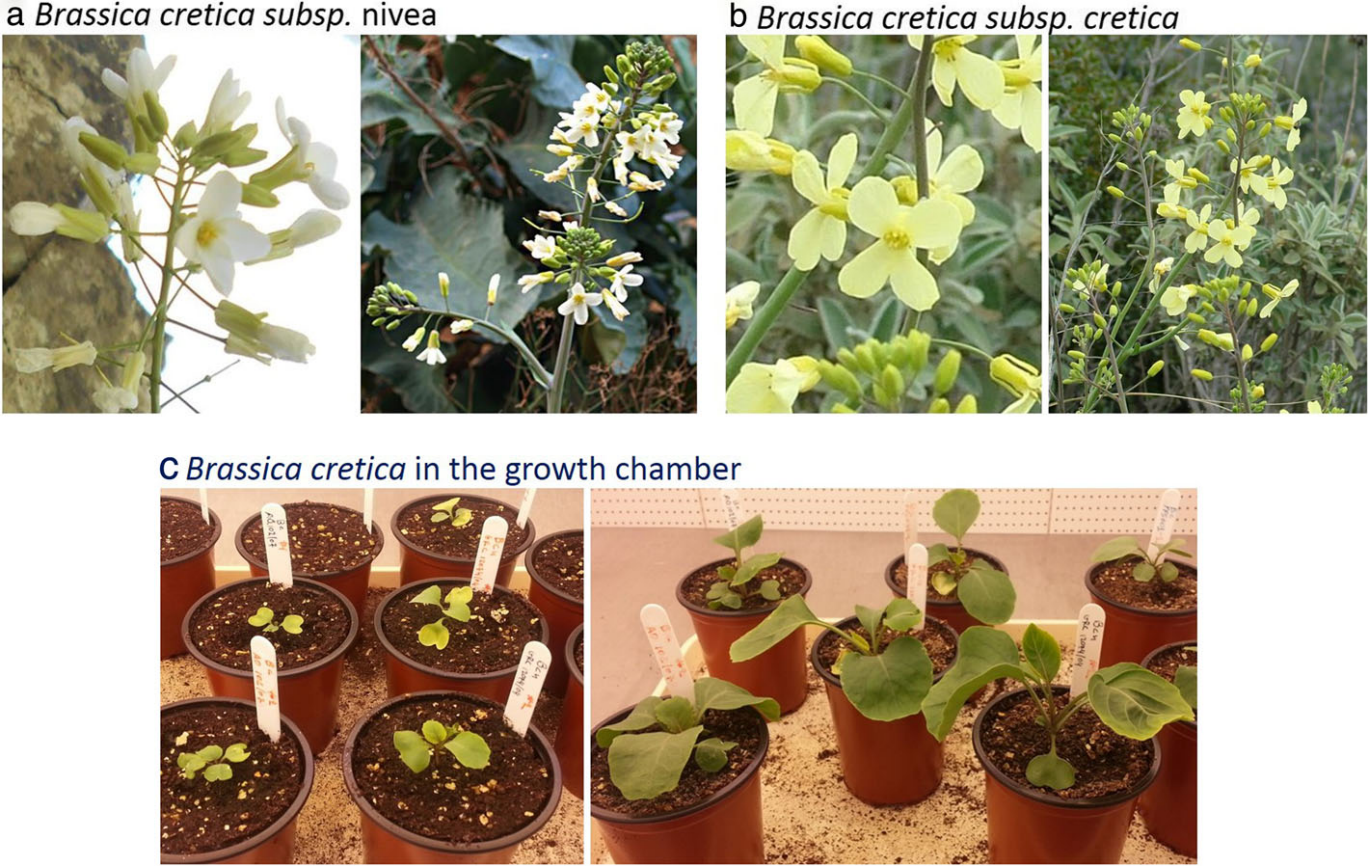
**a**: *Brassica cretica subsp. nivea*; **b:** *Brassica cretica*; **c:** *Brassica cretica* in the plants’ growth chamber

Among the Aegean islands, Crete is the largest and the most floristically diverse. It has experienced a much longer history of isolation compared to the smaller Aegean islands. Over two-thirds of all Greek plant species are found in Crete and it has the greatest proportion of endemic species in the Aegean area [16–18]. Crete was separated from the mainland of Greece around 8 million years ago [16, 19, 20]. For many Cretan plant species suitable habitat is restricted at present to high-altitude areas that are surrounded by a ‘sea’ of low-lying areas acting as dispersal barriers [21]. This includes various chasmophytic plant species, of which *Brassica cretica* Lam. (Fig. 1) is a typical example. It is a wild plant species preferentially inhabiting limestone cliffs and gorges, mainly in Crete but also in the surrounding coastal areas of other Mediterranean countries [22]. A wild relative of the cultivated cabbage (*B. oleracea* L.), *Brassica cretica* [23] is hermaphrodite (has both male and female organs) and pollinated by insects. This CWR species is diploid (2n = 18), partially self-incompatible and has a native distribution in Greece (mainly Crete and North Peloponnese). The plants are perennial and up to 150 cm high, with white or yellow, insect-pollinated flowers that develop into siliqua. Preliminary analyses of electrophoretic variation show that *B. cretica* is outcrossing (little deviation from Hardy-Weinberg equilibrium) and that populations on Crete have undergone extensive divergence at allozyme loci [23]. The geographical isolation has been proposed as the main reason of the significant differences observed among the local *B. cretica* populations for several morphological traits [22, 24]. Furthermore, flower colour differences could constitute an additional mechanism of genetic isolation among populations if different pollinators prefer different types of flower [25]. However, the rates of migration among *B. cretica* populations have not been properly quantified, making it unclear whether the low gene flow alone could explain the population divergence, or whether local adaptation (divergent selection) must be invoked. Widén and colleagues [24] reported that the observed high levels of differentiation at allozyme loci and quantitative traits among Cretan *B. cretica* populations, were consistent with non-adaptive differentiation combined with limited gene flow. However, allozymes may not provide accurate assessments of population structure and gene flow, since, at least one allozyme locus is under diversifying selection in a variety of species [16, 26–28]. Moreover, Edh et al. [16] using nuclear and chloroplast microsatellite markers, studied the differentiation of seven Cretan populations of *B. cretica* and concluded that current patterns of diversification in *B. cretica* mainly result from genetic drift.

*Brassica cretica* Lam. is a wild relative of many crops in the genus *Brassica*, proposed to be one of the ancestors of broccoli, Brussel sprouts, cabbage, cauliflower, kale, swede, turnip and oilseed rape. Since this species is thought to be a gene donor of many crops in the *Brassica oleracea* group, it might contain genes that are not present in the domesticated crops, as well as, a different set of resistance genes (*R* genes) that code for the intracellular immunity NLRs receptors (NOD-Like Receptors). Analysis of the NLRsome of wild species would potentially help us find which genes or loci are responsible for the recognition of effectors from important phytopathogens and thus create resistant plants in the field via transfer of these favored genes/loci [29].

### Aim of this work

Here, we perform genome-wide resequencing of four individuals of *B. cretica* to investigate mechanisms of diversification of four isolated *B. cretica* populations taking into consideration their genomic and subspecies variation. That analysis is based on alignment of sequence data against the reference genome of *B. oleracea* and is not dependent on de novo assembly of the *B. cretica* genome. Nevertheless, we also assembled the sequence data to generate draft assemblies of the four *B. cretica* genomes, which may serve as a useful resource for bioprospecting of traits for introgression into brassicaceous crops.

## Results

### *Genome-wide resequencing of B. cretica*

**Fig. 2:**
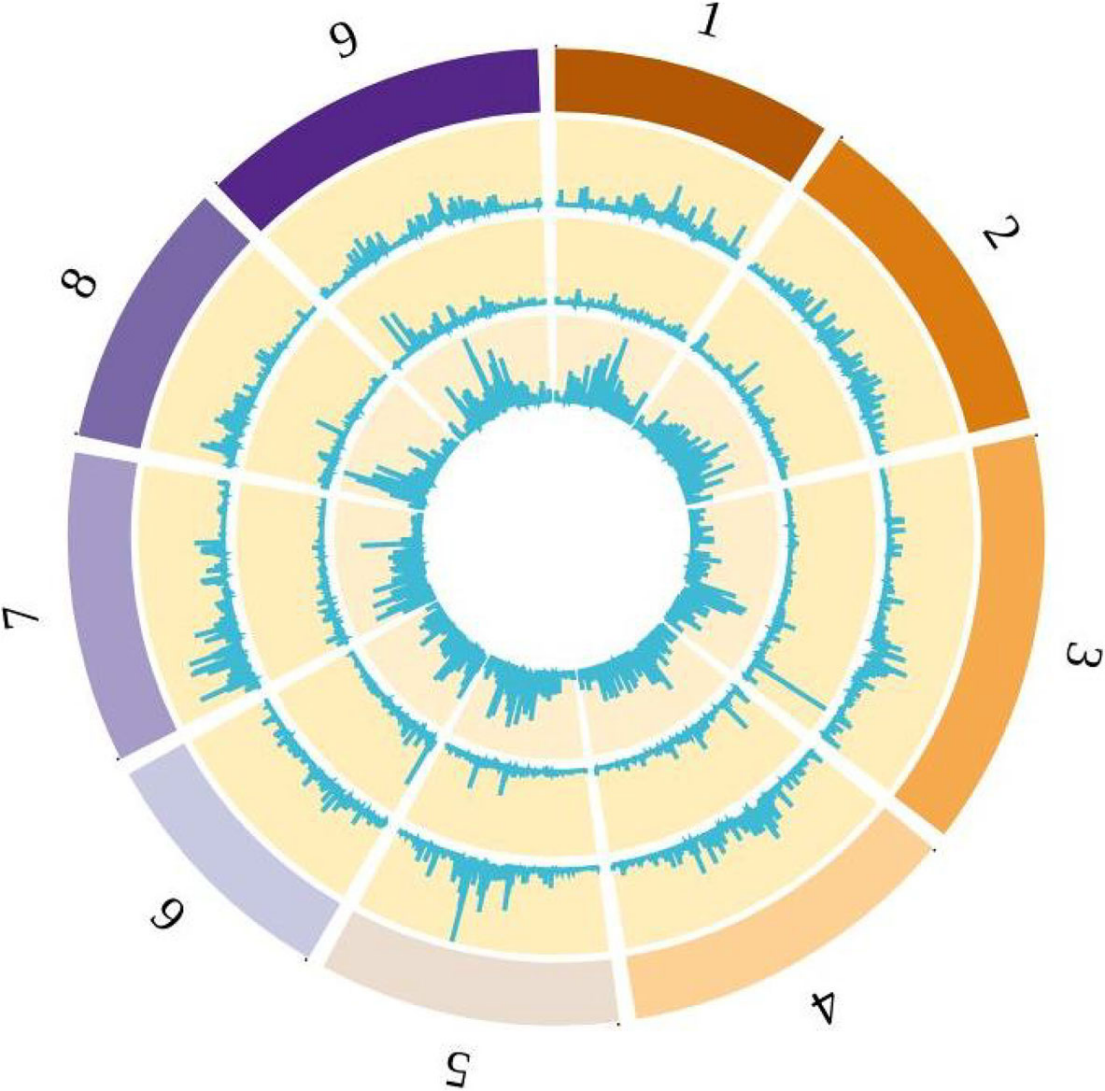
Distribution of variation across the *B. cretica* genomes. Variants were identified by aligning *B. cretica* genome resequencing reads against the *B. oleracea* reference genome as described in the Methods section. The outer ring represents the nine pseudomolecules of the reference sequence. The next ring indicates the density of SNPs that distinguish all four *B. cretica* genomes from the *B. oleracea* reference genome. The next ring represents density of SNPs that distinguish subspecies *nivea* from the other two *B. cretica* genomes. The innermost ring show density of other SNPs that show variation among the four *B. cretica* genomes. The image was rendered using BioCircos [30]

Sequencing of genomic DNA yielded 73.3 M, 83.3 M, 82.4 M and 53.1 M pairs of 300-bp reads respectively from individuals PFS-1207/04, PFS-001/15, PFS-109/04 and PFS-102/07. Aligning these reads against the *B. oleracea* reference genome resulted in 54.8, 62.6, 63.6 and 39.5-fold average depths of coverage respectively. The alignments of resequencing reads versus reference genome were used for variant calling on which the demographic analysis is based (see below). The distribution of variants across the reference genome is summarized as a Circos plot in Fig. 2.

**Fig. 3:**
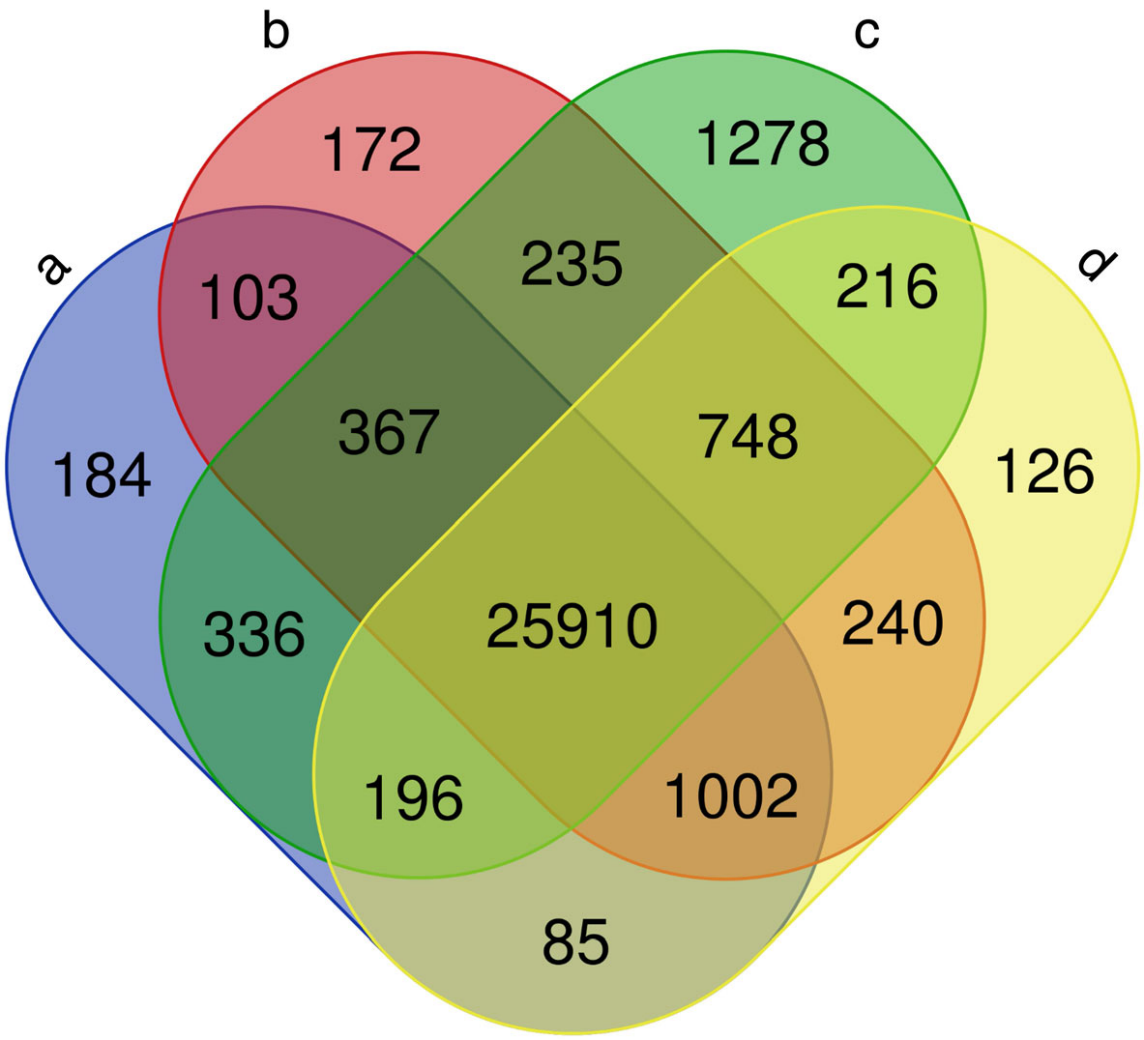
Conservation of *Brassica oleracea* genes across the four re-sequenced genomes of *Brassica cretica*. The Venn diagram shows the numbers of genes confirmed to be present in each of the four genomes. Genes and reference genome sequence were taken from the Bolbase database [31]. Sequence reads were aligned against the reference genome with BWA and coverage of each gene calculated using the *coverageBed* utility in BEDtools [32]. A gene was considered present only if it was 100% covered by sequence reads. The Venn diagram was rendered using the webserver [33]. The full list of coverages for each gene is provided in Additional File 3: Table S5

This genome-wide resequencing data also allowed us to assay conservation of genes among the four *B. cretica* genomes, by examining coverage of annotated genes in the *B. oleracea* reference genome (see Fig. 3). The majority of *B. oleracea* genes are conserved in all four *B. cretica* genomes; however, significant numbers of genes are private to a single individual or subset of the four individuals (Fig. 3; Additional file 3: Table S5).

### Draft genome assemblies

Since no reference genome sequence is available for *B. cretica,* we used the reference genome of the closely related *B. oleracea* for the variant calling that underlies the demographic analysis that is the focus of this study. Nevertheless, our generation of sequence data from *B. cretica* genomes presented the opportunity to assemble draft-quality genome sequences that could be a useful resource for future studies on this CWR species.

We performed *de-novo* assembly using SOAPdenovo2 and deposited these in GenBank under accessions GCA_003260655.1, GCA_003260635.1, GCA_003260675.1 and GCA_003260695.1. These assemblies have limited usefulness, being poorly contiguous, with N_50_ contig lengths of between one and three kilobases; however, they have the advantage of being purely de novo and therefore not dependent on any assumptions based on a reference genome.

To improve contiguity, we subjected the initial assemblies to reference-guided scaffolding against the published *B. oleracea* reference genome using RaGOO [34] and then performed 10 iterations of gap-closing using GapFiller [35] scaffolding. The resulting assemblies were submitted to GenBank as GCA_003260655.2, GCA_003260635.2, GCA_003260675.2 and GCA_003260695.2.

Table 1 shows that reference-guided scaffolding and gap closing generated significantly more contiguous assemblies, with contig N_50_ lengths ranging from 13.4 to 25.9 kb and scaffold N_50_ lengths exceeding 20 Mbp. We also assessed the completeness of gene-space in each of our assemblies and previously published *Brassica* genome assemblies, using BUSCO3 [37]; results are summarized in Table 1. Our *B. cretica* draft genome assemblies are less complete that previously published sequences of closely related *Brassica* genomes. The most complete genome assembly is that of PFS-1207/04, which shows 72% completeness (1534 / 2121). This compares with levels of up to 82% (1752 / 2121) completeness in previously published related genomes (see Table 2). Automated annotation of the PFS-1207/04 genome (GenBank accession GCA_003260655.1) via the MAKER pipeline generated 30,360 predicted protein-coding genes.

**Table 1:** Summary statistics for draft genome assemblies, as assessed by Quast [36]

Assembly	*B. cretica* individual	Contigs	Scaffolds	Contig N_50_ (b.p.)	Scaffold N_50_ (b.p.)	Total length (b.p.)
GCA_003260655.1	PFS-1207/04	106,991	–	2820	–	412,521,210
GCA_003260655.2	PFS-1207/04	22,126	2934	25,920	36,085,697	332,349,879
GCA_003260635.1	PFS-001/15	100,644	–	2197	–	208,353,552
GCA_003260635.2	PFS-001/15	17,704	2393	18,959	21,769,174	208,273,179
GCA_003260675.1	PFS-109/04	108,738	–	1572	–	434,935,090
GCA_003260675.2	PFS-109/04	19,266	2464	26,184	31,308,560	288,739,113
GCA_003260695.1	PFS-102/07	105,350	–	1027	–	40,021,1799
GCA_003260695.2	PFS-102/07	21,300	2337	13,399	21,937,562	202,548,557

**Table 2:** Completeness of gene-space for each genome assembly, as assessed by BUSCO3 (Waterhouse et al. 2017). Each assembly was assessed against the set of 2121 dicotyledonous plant benchmarking universal single-copy orthologs

Assembly	Plant	Complete single copy	Complete duplicated	Fragmented	Missing
GCA_000695525.1 (cite doi: 10.1186/gb-2014-15-6-r77)	*B. oleracea* chromosomes	1741	322	10	48
GCA_000309985.2 (cite DOI: 10.1038/ng.919)	*Brassica rapa*	1705	387	10	19
GCA_000604025.1	*B. oleracea*	1714	378	9	20
GCA_000695525.1 (cite: doi: 10.1186/gb-2014-15-6-r77)	*B. oleracea*	1752	336	10	23
GCA_003260655.1	*B. cretica* PFS-1207/04	1319	171	337	294
GCA_003260655.2	*B. cretica* PFS-1207/04	1534	209	175	203
GCA_003260635.1	*B. cretica* PFS-001/15	619	72	415	1015
GCA_003260635.2	*B. cretica* PFS-001/15	983	99	242	797
GCA_003260675.1	*B. cretica* PFS-109/04	1003	123	457	538
GCA_003260675.2	*B. cretica* PFS-109/04	1275	170	262	414
GCA_003260695.1	*B. cretica* PFS-102/07	658	55	627	781
GCA_003260695.2	*B. cretica* PFS-102/07	1011	89	442	579

### Demographic model inference

Demographic analysis based on genome-wide analysis of genetic variation suggests that populations of *B. cretica* are not isolated. We suggest that the classification of the *B. cretica* in distinct subspecies is not supported by the data. Using only the non-coding part of the data (thus, the parts of the genome that has evolved nearly neutrally), we find the gene flow between different *B. cretica* population is recent and its genomic diversity is high.

**Fig. 4:**
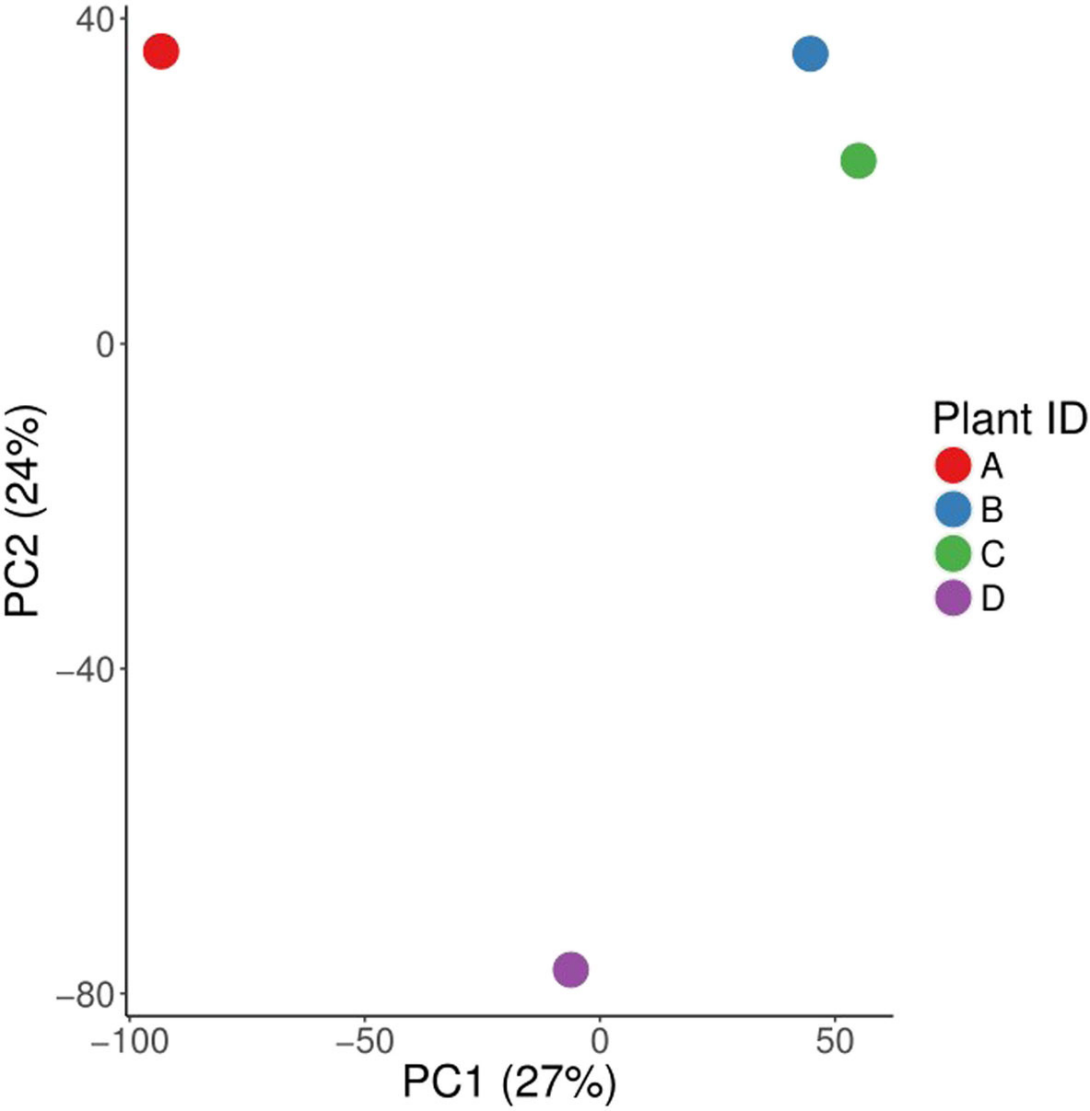
The logPCA results of binary SNP data at the level of the first two Axes. Along the PC1 we defined the members of 2 populations. Population 1 consists of plant A, whereas population 2 of plants B, C, and D. The PC1 and PC2 explain 51% of the data variance

We followed two approaches to infer the neutral demographic model for the *B. cretica* data. The two approaches are related to the separation of the individual plants into distinct groups (i.e., populations or subspecies). According to the first, the subspecies approach, we separate the individuals into two groups specified by their subspecies definition. Plants A and B are characterized as *B. cretica* subsp. nivea SFP1207/94 and *Brassica cretica subsp. nivea* SFP0001/15 (Cretan isolate), respectively, and they constitute group 1, whereas plants C and D are *B. cretica* SFP109/07 and *B. cretica* SFP102/07, respectively, and they define group 2. The second approach is based on the principal component analysis (PCA) plot of the data, which depends on the differences at the DNA level. We call the second approach the genetic approach. We applied logistic principal component analysis (http://arxiv.org/abs/1510.06112v1) (logPCA) since the polymorphisms at each site define a binary state. The results of the logPCA are shown in Fig. 4.

### Demographic model inference based on the subspecies definition

**Fig. 5:**
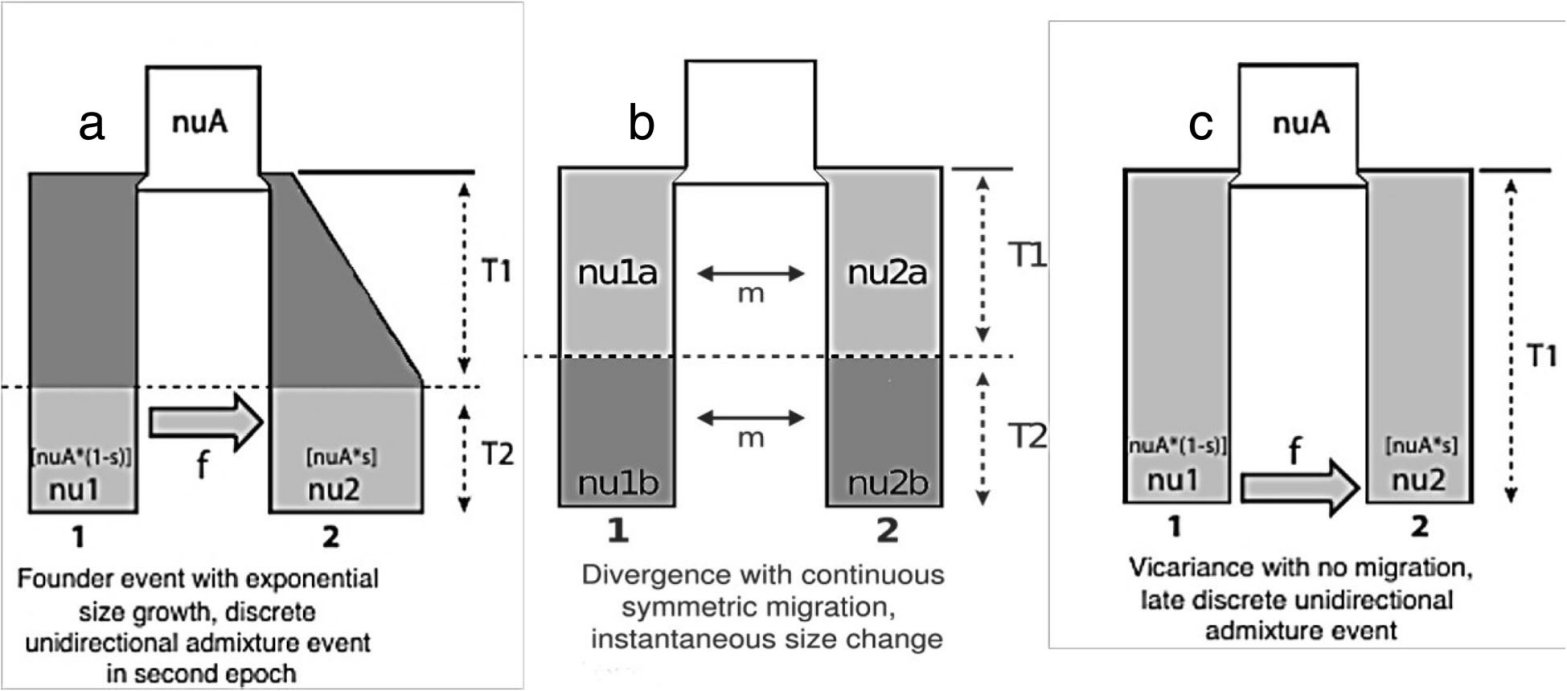
Demographic Model Inference based on the subspecies definition and on the PCA plot, all nu* represent populations with nuA referring to the ancestral population, f signifies the flow for the admixture event, m represents the migration between the populations (units 2N_ref_m_ij_), s is the fraction of the population for each subspecies and T1, T2 represents timepoints for the events (units 2N_ref_). Understanding mechanisms generating parallel genomic divergence patterns among populations is a modern challenge in population ecology, which can widely contribute in the perception of the intraspecific diversification of crop wild relatives. Here we investigated the genomic divergence between three population schemes of *Brassica cretica* using demographic model selection. According to the above results we can support that strict isolation is not recorded between populations. Discrete unidirectional admixture event (**a**) or continuous symmetric migration (**b**) was recorded indicating an absence of insuperable barriers in gene flow between populations. Even in the case of taxonomic segregation (**c**), where strengthen barriers would be expected, late discrete unidirectional admixture event is corroborated

Following the subspecies definition of the two groups of plants, the model “Vicariance with late discrete admixture” is the most likely among the 30 different models with two populations. Such a model suggests that the two subspecies were discrete for a long period of time. However, recently, introgression took place from group 1 (plants A and B) to group 2. Such a massive gene flow suggests that the two groups of plants may not define distinct subspecies, therefore they can be considered as different population of the same species (Fig. 5A).

### Demographic model inference based on the PCA plot

Based on the logPCA results, we identified two populations, the first comprising three individuals (B, C, D) and the second containing one (A). This result is based on the first principal component axis (PC1). It is important to note that although the A, B, and C plants were sampled from Central Greece and D from Crete, logPCA shows that the Cretan individual is genetically closer to B and C than to A. The distances of A and D to the B-C clusters are similar and as a result, we generated an additional population schema grouping together A, B, C and D as another subpopulation, in accordance to the data variability presented along PC2 axis.

**Fig. 6:**
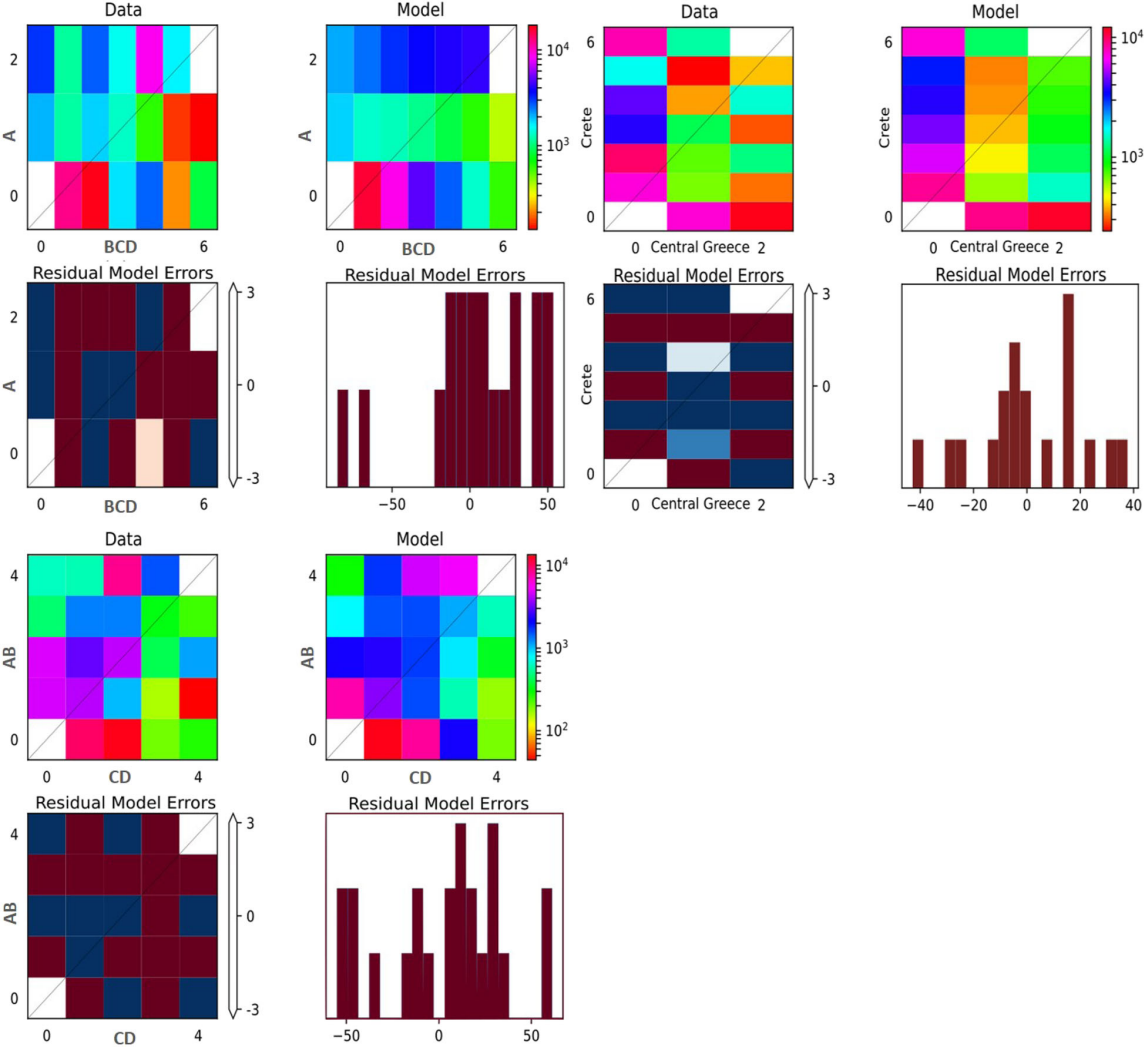
From top right to bottom left: 1) Calculated AFS from *B. cretica* data sets, split by populations. 2) Simulated AFS of the best fitting model from final *dadi* simulations. 3) Heat-map of the residual errors from the comparison between real and simulated AFS. 4) Barplot of the same comparisons

For the first grouping, the “Founder event and discrete admixture, two epoch” model, was selected as the most possible demography model (Fig. 5B). The second grouping resulted in the “Divergence with continuous symmetric migration and instantaneous size change” as the best model to explain the data (Fig. 5C). The first model specifies that the original population split into two subgroups that allowed symmetric migration between them, continuing the population size of each subgroup changed, whereas the second model allows the subpopulations to migrate as the time progresses and the second subpopulation experiences a population size change. The joint 2 population AFS for the real and the simulated data, as well as their difference (residues) are shown in Fig. 6.

In all grouping definitions, it is apparent that populations are not isolated. There is considerable gene flow between all possible groupings of the populations. Especially, in the subspecies-based grouping, the inferred model proposes introgression between the two groups, i.e., massive, directional gene flow. Furthermore, a simulation examination suggests that dadi is able to distinguish a model with gene flow from a model without gene flow in about 79% of the cases indicating a rather robust inference outcome (see Methods). Thus, the subspecies classification scheme of the studied *Brassica cretica* plants may be, in fact, not supported by the modelling outputs. The parameter values for all inferred demographic models as well as the AIC scores of the competing models are presented in the Additional file 2: Tables S1, S2 and S3.

The above finding poses the need for further studies concerning the potential gene flow between populations of *B. cretica* and their effects in adaptive traits in both in situ and ex situ conservation strategies, as well as in cases of genetic improvement especially with newly introduced genes [38].

## Discussion

This study used genome-wide resequencing to investigate mechanisms of diversification of four isolated *B. cretica* populations, taking into consideration their genomic and subspecies variation. There is already evidence of gene flow between wild and crop types of *Brassica* [39]. Similar observations have been reported in the case of wild relatives of rice [40, 41], which further encourage the incorporation of the followed methodology; that is the demographic model selection in the crop wild relatives research. Of course, habitat suitability should also be taken into consideration [40, 42], since ecological factors may also influence the directions and the spatial patterns of gene flow but in the absence of georeferenced data it was necessarily out of scope of the current article. Nevertheless, in future studies a combination of the followed methodology with Ecological Niche Modelling (ENM) [43, 44] is highly recommended.

In the case of taxonomic segregation, the “vicariance-driven divergence with no migration in the early stages” model indicates that the two taxa typically formed as the result of novel and/or emerging geographical barriers, possibly in combination with genetic drift and/or with the contribution of local adaptation for some traits. Concerning whether non-ecological versus ecological process of genetic isolation took place [45], we cannot resort to a single explanation since our data are not adequate for such an inference. The late discrete unidirectional admixture event conforms to the classical view that in different periods in the evolutionary history of a taxon, different factors (ecological and/or non-ecological) may contribute to the process of speciation inducing or failing to complete it [46]. Nevertheless, taking into consideration the prevailing hypothesis that plant diversification in the Aegean region is driven by neutral rather than adaptive differentiation among isolated populations [16, 47–49], we can consider genetic drift as a possible scenario for this population scheme. It is worthy of mention that a few studies using population and landscape genetics approaches in *Brassicaceae* have already revealed a significant signal indicating local adaptation [50]. Smykal et al. [51] also proposed that most of the variation they detected within and between populations of wild pea in northern Fertile Crescent reflects genetic processes such as drift, founder effect and infrequent out-crossing with related individuals, rather than environmental selection pressure.

Unidirectional gene flow has also been reported in other organisms, such as in the case of two lizard subspecies, where gene flow from one subspecies (*Podarcis gaigeae* subsp*. weigandi*) into another (*Podarcis gaigeae* subsp*. gaigeae*) but not in the other direction, recorded by Runemark et al. [52]. In our case, it takes place from the *B. cretica* subsp. *nivaea* into the *B. cretica*. Flower colour might be an explanatory factor of the unidirectional admixture event, as in *B. cretica* subsp. *nivea* it is white, while in *B. cretica* it may vary from white to bright yellow; however, this explanation contradicts Edh et al. [16] who claimed that there is no evidence that flower colour has had in their study any significant effect on gene flow via pollen among the investigated *B. cretica* populations. Nevertheless, in the view of Edh et al. [16] it is depending on the sensitivity of the selected markers (nuclear and chloroplast microsatellites) this flower-coloured based explanation remains standing. Baack et al. [53] report several cases of pre-pollination reproductive isolation related with flower colour and pollinator behavior.

However, independently of whether population genomic divergence is driven by non-ecological or ecological underline mechanisms, the consequences of this late unidirectional admixture event possibly contributed to the high uncertainty or absence of clear consensus of the status of these taxa, as already reported by Edh et al. [16] This is also in line with the treatment of these taxa in the recent Vascular Flora of Greece [54], where the taxon *B. cretica* subsp. *nivea* has not been suggested as a standing subspecies.

In the case of non-taxonomic segregations, that is the case of genomic-variation based population schemes, both divergence and founder event were recorded as split mechanisms of the original population, while continuous symmetric migration and discrete unidirectional admixture event in late epoch respectively were specified. In the literature of population genetics, migration and gene flow are often used interchangeably [55]. Nevertheless, migration refers to the movement and dispersal of individuals or gametes, and gene flow for the movement of alleles, and eventually their establishment, into a genetic pool different from their genetic pool of origin [55, 56]. In our case a more appropriate term to use for migration would be dispersal, as migration is mainly used for animals, incorporating also the seasonal movements.

## Conclusion

In contrast to selection pressure, chance events play a central role in the genomic variation between populations by founder effect [45]. Consequently, in the case of the resulted founder effect demographic model, we can eliminate the role of the environment from consideration as an important contribution to genetic variation, while in the case of the divergence model, the genomic variation may be a result of selection pressure strengthening the role of environment. Nevertheless, despite predictions on the disruptive effect of gene flow in adaptation, when selection is not strong enough to prevent the loss of locally adapted alleles, an increasing number of studies show [55] that gene flow can promote adaptation, that local adaptations can be maintained despite high gene flow, and that genetic architecture plays a fundamental role in the origin and maintenance of local adaptation with gene flow. Thus, in the genomic era it is important to link the selected demographic models with the underlying processes of genomic variation because, if this variation is largely selectively neutral, we cannot assume that a diverse population of crop wild relatives will necessarily exhibit the wide-ranging adaptive diversity required for further crop improvement.

## Methods

### Plant material

Due to the high phenotypic variability of *B. cretica*, a number of subspecies and varieties have been defined. Snogerup et al. [22] recognize three subspecies of *B. cretica*: subsp. *aegea*, subsp. *cretica*, and subsp. *laconica*, whereas Gustafsson et al. [57] suggest only two subspecies, subsp. *cretica* and subsp. *nivea* (sometimes referred to as *B. cretica* subsp. *cretica* var. *nivea* [58];), which includes (pale) yellow and white-flowered variants, respectively.

According to the Vascular Flora of Greece [54] there are three subspecies: *B. cretica* subsp. *aegaea* (Heldr. & Halácsy; Snogerup; Gust & Bothmer), *B. cretica* subsp. *cretica* and *B. cretica* subsp. subsp. *laconica* (Gust. & Snogerup), while *B. cretica* subsp. *nivea* (Boiss & Spruner; Gust. & Snogerup) and *B. nivea* (Boiss & Spruner) are considered as synonyms and misapplied to *B. cretica* Lam. subsp. *cretica*, which has been reported for the mainland of Greece and for the floristic region of Crete and Karpathos [54].

For the present study, three mainland and one island population of *B. cretica* from Greece have been studied. Two *B. cretica* subsp*. nivea* (Boiss & Spruner) M. A. Gust. & Snogerup individuals from the first two mainland populations respectively (A, B) and two *B. cretica* Lam. individuals, one from the third mainland population (C) and the other from Crete, the island population (D), have been used for the genome assemblies (Fig. 1). The studied taxa are not protected by National law or EU legislation. Moreover, the plant species is not included in the Appendices of the Convention on International Trade in Endangered Species of Wild Fauna and Flora (CITES). The plant material was provided by the National Seed Bank of Greece, under the authority of the Greek Ministry of Rural Development and Food.

### Total DNA extraction, library preparation and sequencing

Genomic DNA was extracted from the young emerging leaves using two previously published protocols. For total DNA isolation up to 1 g plant leaf tissue was used. For the DNA isolation we used several protocols including the DNeasy Plant Mini Kit from Qiagen, as the manufactures propose. Likewise, we used a modified triple CetylTrimethyl Ammonium Bromide (CTAB) extraction protocol for total plant DNA isolation, as it has been described before [59].

The yield and quality of DNA were assessed by agarose gel electrophoresis and by a NanoDrop spectrophotometer (NanoDrop Technologies, Wilmington, Delaware) and quantified by Qubit broad range assay (Thermo Fisher Scientific). Illumina sequencing libraries were prepared, after fragmenting 500 ng of DNA to an average size of 500 bp, using NEXTflex 8-barcode Rapid DNAseq kit for Illumina sequencing (Perkin Elmer) with adapters containing indexes and 5–8 cycles polymerase chain reaction (PCR) [60]. Library quality was determined using D1000 screen-tapes (Agilent) and libraries were either sequenced individually or combined in equimolar pools.

Sequencing was performed on the Illumina HiSeq 2500 at the University of Exeter, using a Rapid-Run flowcell, yielding pairs of 300-bp reads.

### Genome assembly

Prior to assembly and alignment, Illumina HiSeq sequence reads were filtered on quality scores and trimmed to remove adapter sequences using Trim Galore [61] with q = 30 (Quality Phred score cutoff = 30). Reads were assembled into contigs using SOAPdenovo2 [62] with k = 127 (k-mer value = 127). Configuration files used for the SOAPdenovo2 assembly can be found on FigShare at under DOI 10.6084/m9.figshare.7583396. Contigs shorter than 500 bp in length were removed.

### Variant calling

#### We used the closely related species

After trimming and filtering with TrimGalore, sequence reads were aligned against the reference sequence using Burrows-Wheeler Aligner (BWA) [63] mem version 0.7.15-r1140 with default options and parameter values. Candidate SNVs were identified using Sequence Alignment/Map tools (SAMtools)/binary call format tools (BCFtools) package, version 1.6 [64], using the following command-lines:

samtools mpileup -u -f genome.fasta alignment.bam 4 alignment.bcf and *Brassica oleracea* as reference to map the contigs from the four plants using the Burrows-Wheeler Aligner [65]. The produced SAM files were then converted to BAM by samtools [64]. Using the BAM files, we marked the duplicates and called variants per-sample using Haplotype Caller as indicated by the GATK Best Practices. We followed the pipeline to create a single VCF file identifying the joined-called SNPs and indels which are ready for filtering. Concluding the GATK pipeline, we filtered the variants by quality score recalibration. We transformed the final VCF file to ms [66] output since the dadi python package requires ms format by using a custom script, (Fig. 2 for the distribution of these variants across the genome).

### Genome annotation

Genome annotation was performed using the MAKER pipeline [67, 68] version 2.31.10. Ab initio gene prediction was performed using Augustus [69] version 3.1 trained on *Arabidopsis*. Configuration files for the MAKER annotation can be found on FigShare under DOI 10.6084/m9.figshare.7583672. The GFF file generated by MAKER was converted into NCBI’s Feature Table (.tbl) format using Genome Annotation Generator [70] version 2.0.1.

### Allele frequency Spectrum (AFS)

The AFS defined as ξ = {ξ_i_: number of sites with derived allele counts being *i*} is a useful summary of the data especially for demography inference. To calculate the AFS, we mapped the reads of *B. cretica* to the *B. oleracea* reference genome. This allowed us to use all specimens and also to use the *B. oleracea* as an outgroup that denotes the ancestral state. Following the GATK best practices pipeline [71], this mapping resulted in approximately six million single nucleotide polymorphisms (SNPs).

*Brassica oleracea* has been examined thoroughly in the past and there is a gene list of the organism organized into chromosomes. We used this list to exclude SNPs with a distance less than 10 kb from those coding regions. This process of removing SNPs is necessary when the SNPs are used to infer the demographic model. Due to linkage disequilibrium SNPs within or in the proximity of genic regions are affected by selection forces, especially negative selection. Negative selection effectively increases the low frequency derived variants and therefore it introduces biases in the demographic inference. For this reason, we excluded SNPs located within or in the proximity of genic regions.

### Demographic inference

#### Inferring the demographic model employing genome-wide data

Reconstructing the demographic history of a population is a process based on statistical inference. The amount of available information is therefore critical for the robust inference of the demographic model. Analyzing a small number of non-recombining loci, even with large sample size (number of individuals) results in poor inference because the power fades rapidly upon moving back in time and only a few independent lineages remain (coalescent rate is related to the square of the number of lineages). A better approach is to use genome-wide data even with a small number of individuals. Thus, in contrast to the many-individuals approach, a few genomes (even a single diploid genome) contains hundreds of thousands of independent loci (due to recombination), each of which provides information about the demographic history of the population. It has been shown that it is possible to infer the demographic history of a whole population even by using a single diploid individual [72].

##### Using dadi to infer the demographic model

Inferring a demographic model consistent with a particular data set requires random walks into a large parameter space by simulating the model using Monte Carlo coalescent-theory based approaches. The most well-known approach based on Monte Carlo coalescent simulations is the Approximate Bayesian Computation (ABC) inference [73]. The main handicap of these methods is their scalability to genome-wide size data sets. Another issue arises when multiple populations are free to interact through migration (either symmetric or asymmetric) resulting in an increase of the parameters and, therefore, the required complex calculations. These complexities hinder any effort to thorough explain the statistical properties of the summary statistics produced during the walks. To avoid these problems we based our demographic model inference on the multi-population allele frequency spectrum (AFS) [74–77], due to the fact that demographic history of a population is reflected in the allele frequency spectrum. By comparing the different spectra produced by simulations and observations we can access the model’s goodness of fit and estimate the best parameter values for each model.

In spite of the existence of efficient algorithms for the simulation of a single population AFS [78–80], the joint AFS between two or more populations still requires very computationally intensive coalescent simulations. For more than two populations the computational complexity becomes prohibitively large. Approximations of the joint-AFS using a numerical solution of a diffusion equation have been used extensively in the past [81], enabling simulations of a joint-AFS for two populations in a reasonable computation time. Although the diffusion approach neglects linkage disequilibria, we can use composite likelihood function as a consistent estimator for evaluating genetic scenarios. Concerns about the use of composite likelihood in population genetics are overcome by allowing conventional and parametric bootstrap of the data.

The *dadi* python package [82] implements these approximations and in conjunction with the *dadi_pipeline* described in [83] allows for adequate exploration of the parameter space. The *dadi_pipeline* consists of three optimization rounds and a final plotting step. We used 30 demography models ranging from simple (populations never diverge) to complex (ancient divergence with asymmetric migrations between the two populations) to find the best fitting model. These demographic models comprise a thorough list of two population possible models and they were first examined by Portik et al. [83].

The initial two rounds of optimizations search the parameter space for the parameter set that best describes the data under each of the thirty models. For every model we sampled 50 different parameter sets and 50 repetitions of the each set to get the actual global maximum for each model while avoiding local maxima. We based our selections of the best parameter values on the AIC score for each model. To assess which demographic model better reflects the true demographic history of the *B. cretica* population a simple comparison between the respective AIC scores from each model is not valid because AIC is not comparable between non-nested models. We compared the models using Akaike weights [84], by calculating the difference between each model’s AIC and the AIC of the best candidate model. With a simple transformation we can calculate an estimate of the relative likelihood L_i_ of each model *i* and by dividing each Li with the sum of Li we can normalize the weights and compare the models, and therefore we can find the model that better fits the data [84].

##### Dadi pipeline performance on small sample sizes

The dadi_pipeline was successfully used in the past for identifying the demography model of populations with more than 8 samples [83]. To estimate its performance on our number of samples, we chose the A-BCD grouping and simulated 100 datasets using Hudson’s ms [66] given our proposed parameters as arguments.

We run the three optimization rounds of the pipeline for each dataset, using our proposed model and a model that specifies no gene flow between the populations.

We used the Akaike Information Criterion (AIC) to compare the fit of a model with gene flow and a model without gene flow.. We subtracted the AIC of the gene flow model from the AIC of the no gene flow model. A positive result indicates that dadi correctly identifies our proposed model with gene flow as the better fit for the simulated data. The dadi_pipeline is successful in this task in 79% of the cases (Additional file 1: Fig. S1).

## Supplementary information

**Additional file 1: Figure S1.** Differences of AIC between the no gene flow model and the proposed (gene flow) model.

**Additional file 2: Table S1A.** Top 3 AIC relative weights models with BCD-A cluster. **Table S1B.** Parameters of optimal model on BCD-A cluster. **Table S2A.** Top 3 AIC relative weights models with ABC-D clusters. **Table S2B.** Parameters of optimal model on ABC-D cluster. **Table S3A.** Top 3 AIC relative weights models with the AB-CD clusters. **Table S3B.** Parameters of optimal model on AB-CD clusters.

**Additional file 3: Table S5.** The full list of coverages for each gene.

## Data Availability

All genome sequence assemblies and genomic sequence reads are freely available from GenBank and the Sequence Read Archive respectively under BioProject accession PRJNA470925. The GenBank accession numbers for the assemblies are: *Brassica cretica* PFS-1207/04: GCA_003260655.1 & GCA_003260655.2; *Brassica cretica* PFS-001/15: GCA_003260635.1 & GCA_003260635.2; *Brassica cretica* PFS-109/04: GCA_003260675.1 & GCA_003260675.2; and *Brassica cretica* PFS-102/07: GCA_003260695.1 & GCA_003260695.2.

## Abbreviations

AFS: Allele frequency spectrum
AIC: Akaike information criterion
B: Brassica
CTAB: Cetyltrimethyl ammonium bromide
CWR: Crop wild relatives
ENM: Ecological niche modelling
MY: Million years
NLRs: Nucleotide-binding domain leucine-rich repeat
PCA: Principal component analysis
PCR: Polymerase chain reaction
SNP: Single nucleotide polymorphism
SNVs: Single nucleotide variants
