# Supplementary figures and images for: Intraspecific diversification of the crop wild relative Brassica cretica Lam. using demographic model selection
Source: BMC Genomics. 2020 Jan 14;21:48. doi: 10.1186/s12864-019-6439-x (PMC6961386; doi:10.1186/s12864-019-6439-x)

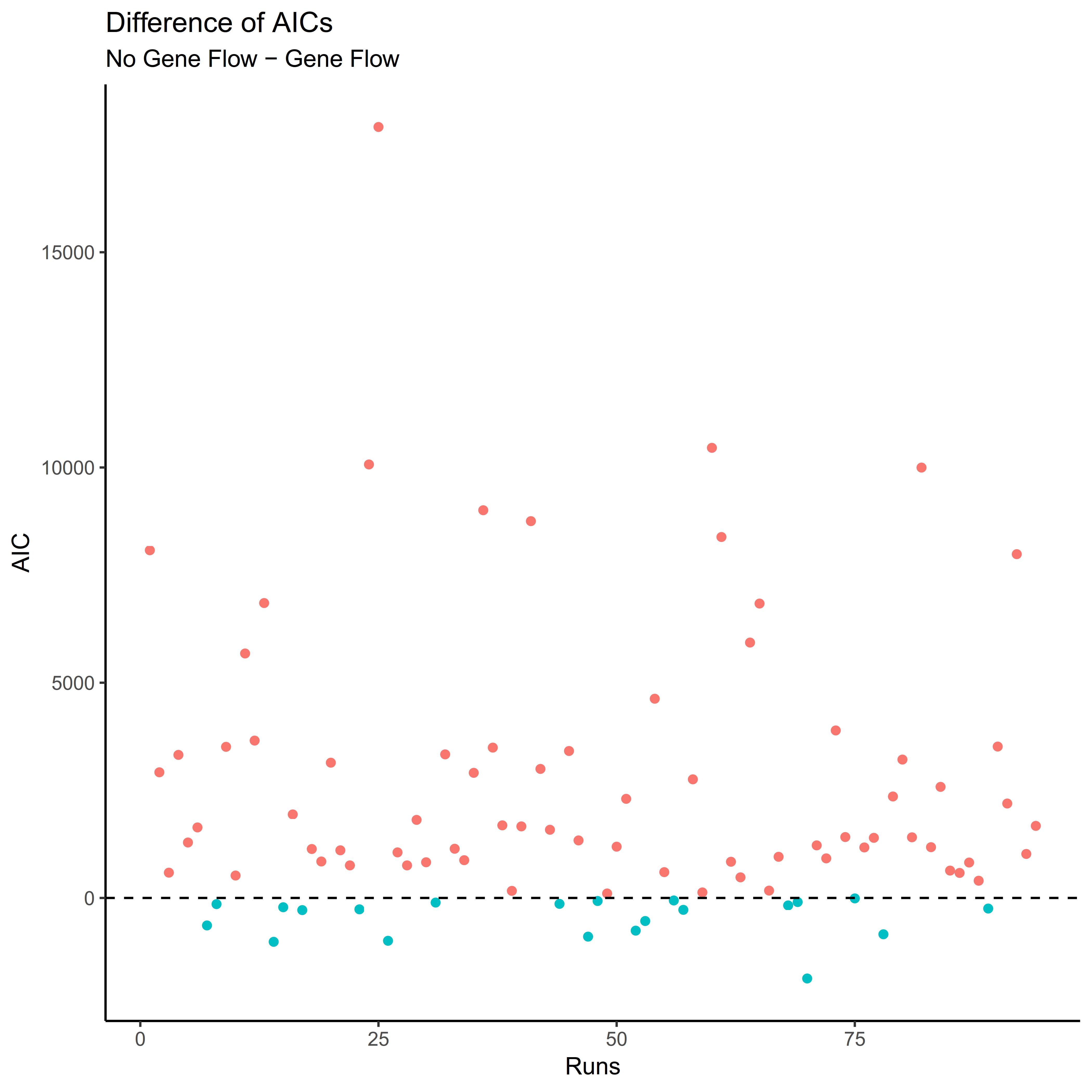

Supplement: Supplementary file 1 — Additional file 1: Figure S1. Differences of AIC between the no gene flow model and the proposed (gene flow) model. [file 12864_2019_6439_MOESM1_ESM.tif]
