# Supplementary material for: Intraspecific diversification of the crop wild relative Brassica cretica Lam. using demographic model selection
Source: BMC Genomics. 2020 Jan 14;21:48. doi: 10.1186/s12864-019-6439-x (PMC6961386; doi:10.1186/s12864-019-6439-x)
Supplement: Supplementary file 2 — Additional file 2: Table S1A. Top 3 AIC relative weights models with BCD-A cluster. Table S1B. Parameters of optimal model on BCD-A cluster. Table S2A. Top 3 AIC relative weights models with ABC-D clusters. Table S2B. Parameters of optimal model on ABC-D cluster. Table S3A. Top 3 AIC relative weights models with the AB-CD clusters. Table S3B. Parameters of optimal model on AB-CD clusters. [file 12864_2019_6439_MOESM2_ESM.docx]

**Supplementary Material**

**Supplementary Table S1A:** Top 3 AIC relative weights models with BCD-A cluster.

| **Model Name** | Relative AIC Weight |
| --- | --- |
| **Founder event and discrete admixture** | ~1 |
| **Divergence with ancient symmetrical migration** | 1.67e-91 |
| **Divergence with no migration** | 5.08e-211 |

**Supplementary Table S1B:** Parameters of optimal model on BCD-A cluster.

| **Ancient population size (nuA)** | 3.3134 |
| --- | --- |
| **Final size of first population** | 1.0835 |
| **Final size of second population** | 0.4178 |
| **Time in the past of split (in units of 2*Na generations)** | 0.0519 |
| **The scaled time between the admixture event and present.** | 4.1887 |
| **Fraction of ancient population that goes to second population. (Pop 1 has size nuA*(1-s).)** | 0.9789 |
| **Fraction of updated population 2 to be derived from population 1.** | 0.3876 |

**Supplementary Table S2A:** Top 3 AIC relative weights models with ABC-D clusters.

| **Model Name** | Relative AIC Weight |
| --- | --- |
| **Divergence with symmetric migration, size change** | ~1 |
| **Vicariance with late discrete admixture** | 9.59e-20 |
| **Founder event and discrete admixture, two epoch** | 4.11e-75 |

**Supplementary Table S2B:** Parameters of optimal model on ABC-D cluster.

| **Size of first population after split** | 2.6648 |
| --- | --- |
| **Size of second population after split.** | 1.8109 |
| **Size of first population after time interval.** | 0.4665 |
| **Size of second population after time interval.** | 0.4633 |
| **Migration rate between populations (2*Na*m)** | 0.0796 |
| **Time in the past of split (in units of 2*Na generations)** | 7.6229 |
| **Time of population size change.** | 0.182 |

**Supplementary Table S3A:** Top 3 AIC relative weights models with the AB-CD clusters.

| **Model Name** | Relative AIC Weight |
| --- | --- |
| **Vicariance with late discrete admixture** | ~1 |
| **Divergence and asymmetrical secondary contact, size change** | 3.23e-694 |
| **Founder event and discrete late admixture** | 4.69e-908 |

**Supplementary Table S3B:** Parameters of optimal model on AB-CD clusters.

| **Ancient population size** | 0.4294 |
| --- | --- |
| **Fraction of ancient population that goes to the second population. (First population has size nuA*(1-s).)** | 0.0174 |
| **Size of first population after split.** | 0.6607 |
| **Size of second population after split.** | 0.0889 |
| **Time in the past of split (in units of 2*Na generations)** | 0.6899 |
| **Fraction of updated second population to be derived from first population** | 0.6405 |
